# Genome-wide association study and its applications in the non-model crop *Sesamum indicum*

**DOI:** 10.1186/s12870-021-03046-x

**Published:** 2021-06-22

**Authors:** Muez Berhe, Komivi Dossa, Jun You, Pape Adama Mboup, Idrissa Navel Diallo, Diaga Diouf, Xiurong Zhang, Linhai Wang

**Affiliations:** 1 Oil Crops Research Institute of the Chinese Academy of Agricultural Sciences, Key Laboratory of Biology and Genetic Improvement of Oil Crops, Ministry of Agriculture, and Rural Affairs, No.2 Xudong 2nd Road, Wuhan, 430062 China; 2 Humera Agricultural Research Center of Tigray Agricultural Research Institute, Humera, Tigray Ethiopia; 3 Laboratoire Campus de Biotechnologies Végétales, Département de Biologie Végétale, Faculté des Sciences et Techniques, Université Cheikh Anta Diop, BP 5005 Dakar-Fann, 10700 Dakar, Senegal; 4 Laboratory of Genetics, Horticulture and Seed Sciences, Faculty of Agronomic Sciences, University of Abomey-Calavi, 01 BP 526, Cotonou, Republic of Benin; 5 Département de Mathématiques et Informatique, Faculté des Sciences et Techniques, Université Cheikh Anta Diop, BP 5005 Dakar-Fann, 10700 Dakar, Senegal

**Keywords:** GWAS, Sesame, Statistical models, Genomics assisted breeding

## Abstract

**Background:**

Sesame is a rare example of non-model and minor crop for which numerous genetic loci and candidate genes underlying features of interest have been disclosed at relatively high resolution. These progresses have been achieved thanks to the applications of the genome-wide association study (GWAS) approach. GWAS has benefited from the availability of high-quality genomes, re-sequencing data from thousands of genotypes, extensive transcriptome sequencing, development of haplotype map and web-based functional databases in sesame.

**Results:**

In this paper, we reviewed the GWAS methods, the underlying statistical models and the applications for genetic discovery of important traits in sesame. A novel online database SiGeDiD (http://sigedid.ucad.sn/) has been developed to provide access to all genetic and genomic discoveries through GWAS in sesame. We also tested for the first time, applications of various new GWAS multi-locus models in sesame.

**Conclusions:**

Collectively, this work portrays steps and provides guidelines for efficient GWAS implementation in sesame, a non-model crop.

**Supplementary Information:**

The online version contains supplementary material available at 10.1186/s12870-021-03046-x.

## Background

Sesame (*Sesamum indicum* L, 2n = 2x = 26) which belongs to the *Pedaliaceae* family is one of the most ancient oilseed crops domesticated from the wild progenitor *S*. *malabaricum* in Near East, Asia and Africa over 5,000 years ago [1, 2]. Sesame is reputed for its climate-resilience, high oil content, and unique antioxidant properties [3]. It is an important source of high-quality edible oil and protein food. The oil content of sesame seed ranges from 50-60% with a high proportion of natural antioxidants such as sesamolin, sesamin, and sesamol, conferring a long shelf life and stability to the oil [4, 5]. Ashakumary et al. [6] reported that sesame seed contains 19-25% protein and is a good source of iron, magnesium, copper, calcium, vitamins B1, E and phytosterols that help to lower the levels of blood cholesterol. Besides, all essential amino acids and fatty acids are present in the sesame seed [7]. The sesame sector is a billion-dollar industry that supports the livelihoods of millions of farmers throughout the world [8]. The total production has significantly increased over the last ten years, reaching 6 million tons in 2017 (Food and Agriculture Organization Statistical Database [9]. Sesame production and productivity, however, face different constraints, including limited numbers of improved varieties, shattering of capsules at maturity, non-synchronous maturity, poor stand establishment, profuse branching, low harvest index, drought stress, waterlogging and diseases [10–12]. To accelerate sesame improvement, genomics assisted breeding has been adopted as an efficient approach for developing superior varieties in a short time [13]. Hence, the reference genome sequence of sesame together with numerous essential genomic resources was delivered to the scientific community [14]. The haplotype map of the sesame genome was constructed from a re-sequencing project of 705 worldwide diverse cultivars and two representative genomes were further *de novo* assembled [15]. These resources are vital to the quick advancement of sesame research, as they expedite the detection of genetic loci that control important agronomic traits using the genome-wide association study (GWAS) approach. Today, hundreds of causative genetic variants associated with important traits such as oil quality, abiotic stress resistance, seed yield have been discovered. These findings facilitate the use of marker-assisted selection and genomic selection to advance genetic improvement and overall productivity of sesame. This makes sesame a rare case of non-model and minor crop for which genomic studies, particularly GWAS, have been very successful.

In this review paper, we first present the GWAS approach and underlying statistical models. Then, the ongoing efforts of genetic discovery through applications of GWAS in sesame are presented in detail. We conclude this paper with important guidelines for better applications of GWAS in sesame.

## Main text

### GWAS approach, underlying statistical models and applications in plants

#### GWAS approach

Genome-wide association study (GWAS) also known as association mapping or linkage disequilibrium (LD) mapping takes the full advantage of high phenotypic variation within a species and the high number of historical recombination events in the natural population. It has become an alternative approach over the conventional quantitative trait locus (QTL) mapping to identify the genetic loci underlying traits at a relatively high resolution [15]. GWAS in general is applicable to study the association between single-nucleotide polymorphisms (SNPs) and target phenotypic traits. Nowadays, SNP identification is becoming much easier using advanced high throughput genotyping techniques. GWAS, quantitatively is evaluated based on LD by genotyping and phenotyping various individuals in a natural population panel. Unlike the traditional QTL mapping approach, which makes the use of bi-parental segregating populations, identification of causal genes for traits of interest in GWAS is performed in natural populations. A key advantage of GWAS is that the same genotyping data and the same population can be used over and over for different traits.

GWAS has been successfully applied to identify associations at a high resolution, detect candidate genes and dissect the quantitative traits in human, animals, and plants [16, 17]. GWAS in various economically valuable crops has been used to gain insight into the genetic architecture of important traits, including days to heading, days to flowering panicle architecture, resistance to rice yellow mottle virus, fertility restoration, and agronomic traits in rice [18–21]; pattern of genetic change and evolution [22, 23], compositional and pasting properties [24], stalk biomass [25] and leaf cuticular conductance [26] in maize; plant height components and inflorescence architecture [27], grain size [28] and grain quality [29] in sorghum; harvest index in maize [30], flowering time in canola [31], stress tolerance, oil content and seed quality [32] in brassica; oil yield and quality [15], yield related traits [33, 34], drought tolerance [35], vitamin E [36] in sesame.

### Statistical models underlying GWAS approach

#### Single-locus models

Marker-trait association using GWAS has been widely detected using one-dimensional genome scans of the population [19, 37–39]. In this method, one SNP is evaluated at a time. Following the use of general linear model (GLM) which is described as **Y** = **β**_**0**_ + **β**_**1**_**X** [40] (where Y = dependent/predicted/ explanatory/response variable, **β**_**0**_ **=** the intercept**; β**_**1**_ **=** a weight or slope (coefficient); **X =** a variable), a popular model referred as a Mixed Linear Model (MLM) (Q+K method) which is described as **Y** = **Xβ** + **Zu + e** [41], (where **Y =** vector of observed phenotypes; **β =** unknown vector containing fixed effects, including the genetic marker, population structure (Q), and the intercept; **u =** unknown vector of random additive genetic effects from multiple background QTL for individuals/lines; **X** and **Z** = known design matrices; and **e =** unobserved vector of residuals) was developed to control the multiple testing effects and bias of population stratification in GWAS. Then, the accuracy of association mapping has been reported partially improved [17, 42, 43]. Subsequently, numerous advanced statistical methods based on the MLM have also been suggested to resolve certain limitations such as false-positive rates, large computational consequences, and inaccurate predictions [44]. Efficient mixed model association (EMMA) [45], compressed mixed linear model (CMLM) and population parameters previously determined (P3D) [46], and random-SNP-effect mixed linear model (MRMLM) [47] are some of the latest improved single-locus genome scans MLM-based approaches proposed so far. Such advanced statistical models are powerful, flexible, and computationally efficient. EMMA was proposed to minimize the computational load exhibited in the MLM probability functions by considering the quantitative trait nucleotide (QTN) effect as a fixed effect [17, 44, 45]; while CMLM was proposed to control the size of huge genotype data by grouping individuals into groups and, thus, the group kinship matrix is derived from the clustered individuals [46]. Generally, despite its limitation for efficient estimation of marker effects in complex traits, the single-locus model approach has a good ability to handle several markers [47], and this is one of its worthy reported features.

Although the single-locus model analysis was a common approach for association analysis between each SNP and phenotype in GWAS, some earlier reports suggested that the use of a single-locus model analysis has limitations to resolve potential effects caused by multiple tests, historical genotype effects and pleiotropic effects [17, 48]. They reported that the interaction between the available genetic variants throughout the genome is not profoundly explored when only on SNP is tested at a time. Similarly, the Bonferroni correction employed to control the false-positive error (FDR) due to multiple testing is also very stringent in this approach, hence significant numbers of important loci may not be identified by the single-locus models particularly for large errors due to phenotypic data and multi-locus effects [49, 50]. Thus, it has been suggested that these single-locus genome scan methods are not convenient to test quantitative traits regulated by a few and/or many genes with large and minor effects, respectively [17, 49]. Besides, the genetic epistatic effects generated within close genes could not be explored in single-locus methods [51].

#### Haplotype-based models

To address some of the limitations in the single-locus model analysis, haplotype-based models, which is conducted based on a random SNP effect mixed linear model (MRMLM) described as: **Y =Xβ + Z**_**k**_**y**_**k**_ **+ u + e** (where **Y** = a vector of estimated genotypic value for all lines is an incident matrix for fixed effects as population structure, **β** is a vector of the fixed effect, **Z**_**k**_ **=** a vector of genotype indicators for k^th^ SNP, **Y**_**k**_ = random effect of marker k with ~N (0, Kσ^2^_k_), **u**= vector of polygenic effects described by the kinship matrix (K) with ~N (0, σ^2^_a_) and **e** = vector of residuals errors with ~N (0, Iσ^2^_e_)), was developed and implemented for some major crops such as wheat, rice, and soybean [52, 53]. Several neighboring markers in high LD are clustered into a single multi-locus haplotype in this multivariate method, thus the haplotypes are evaluated in a multiple GLM system rather than individual SNPs, and the associations between the haplotypes and the traits under selection have been observed [48, 52, 54]. The haplotype-based model is relatively more efficient and reliable than the traditional single-locus models in GWAS as it helps to accurately capture the allelic diversity, optimize the use of high-density marker data, enhance the power of epistatic interactions discovery and minimize multiple testing [51, 52].

#### Multi-locus models

Multi-locus models are newly developed alternative methods in GWAS involving two-stage algorithms [55–57] consisting of a single locus scan of the entire genome to detect all possible associated SNPs (QTNs) and then testing all associated SNPs using a multi-locus GWAS model to detect true QTNs. These newly developed multi-locus GWAS models are ideal for testing complex quantitative traits regulated by multiple genes/loci and less influenced by population structure. Some advantages of multi-locus models over single-locus models are for example, the detection of multiple genes governing a given trait with high power and efficiency, low false-positive rate and no need of Bonferroni correction for multiple testing known to potentially exclude important loci [17, 47, 58, 59]. Multi-locus models have also resulted in substantial improvements of the quality and depth of the association results in GWAS [17, 42, 53, 57, 60, 61]. The models currently largely implemented in GWAS include a multi-locus mixed model (MLMM) [57], multi-locus random SNP-effect mixed linear model (mrMLM) [47], integrative sure independence screening expectation-maximization Bayesian least absolute shrinkage and selection operator model (ISIS EM-BLASSO) [50], fast multi-locus random-SNP-effect efficient mixed model association (FASTmrEMMA) [17], polygene-background-control-based least angle regression plus Empirical Bayes (pLARmEB) [62], Kruskal-Wallis test with empirical Bayes under polygenic background control (pKWmEB) [58] and fast multi-locus random-SNP-effect mixed linear model (FASTmrMLM) [59, 63]. Among the numerous multi-locus models recorded to date, Segura et al. [57] proposed a MLMM method which has an advantage over other existing multi-locus methods, including penalized logistical regression [64], Stepwise regression [65], Bayesian-inspired penalized maximum likelihood, computational efficiency, false discovery rate detection and addressing the problems of population structure in GWAS. Similarly, Korte et al. [66] also proposed a mixed model method referred to as a multi-trait mixed model (MTMM) that detects the causal loci for precisely correlated multiple phenotype traits and simultaneously deals with both intra-trait and inter-trait variance components. Likewise, Klasen et al. [61] suggested a Quantitative Trait Cluster Association Test (QTCAT) analysis of multi-locus associations without employing population correction techniques and this model showed better results in limiting the false positive/negative associations due to correction strategies to mitigate confounding impacts. Multi-Trait Analysis of GWAS (MTAG) was also another specific approach developed by Turley et al. [67] to analyze summary statistics (meta-analysis) in GWAS. Zhan et al. [68] also proposed another method, named Dual Kernel Association Test (DKAT) that includes two individual kernel matrices to explain phenotype and genotype similarities. Some of DKAT's advantages over existing methods include being able to test the relationship between multiple traits and multiple SNPs without making parametric assumptions, correcting Type I error rates, being statistically highly efficient and computationally scalable [60, 68].

Recently, different comparative studies have been conducted to assess the capacity of these different GWAS models in detecting marker-trait associations in different plant species. Globally, it has been found that the multi-locus models were more efficient and powerful than the single-locus models to detect highly significant association results for the traits of interest (Table 1). However, integrating both single-locus and multi-locus models have been proved to enhance the power and validity of the association analysis of complex traits in GWAS because single-locus models could detect some loci that multi-locus models fail to identify [54, 70].

**Table 1:** Comparison of power and efficiency of single and multi-locus models in GWAS for the detection of marker-trait associations

Species	Sample size	Traits under study	Number of traits measured	SNPs number	Statistical Models	Number of models	Maximum QTN detected by each model	Co-detected QTN	Outperformed model	Recommended approach	References
**Arabidopsis**	188	Flowering time	6	216,130	Single-locus	2	25	NA	mrMLM	Multi-locus	[47]
					Multi-locus	1	120				
	199	Flowering time	4	216,130	Single-locus	4	21	NA	FASTmrEMMA	Multi-locus	[17]
					Multi-locus	2	68				
**Maize**	144	Embryonic callus regenerative capacity	5	43427	Single-locus	1	1	63	ISIS EMBLASSO	Multi-locus	[69]
					Multi-locus	4	160				
	230	Starch pasting properties	7	145,232	Single-locus	1	7	7	FASTmrEMMA,	Integrated	[70]
					Multi-locus	3	29				
**Cotton**	160	Fiber quality	6	72,792	Single-locus	1	NA	70	NA	Integrated	[71]
					Multi-locus	6					
	169	Fiber quality	5	53,848	Single-locus	3	342	15	multi-locus	Integrated	[54]
					Multi-locus	3					
**Soybean**	368	Plant height and number of models	6	62,423	Single-locus	1	24	NA	mrMLM	Multi-locus	[72]
					Multi-locus	1	64				
**Wheat**	182	Free amino acid level	20	14,646	Single-locus	1	4	66	pkWmEB	Integrated	[73]
					Multi-locus	6	117				
**Rice**	478	Salt-tolerance	5	165,529	NA	6	NA	56	ISIS EM-BLASSO	Integrated	[55]
					Multi-locus		371				

#### Use of pan-genome vs single reference genome for GWAS

The common approach to study a given population’s genetic variation relies on the interpretation of genes and variants annotated from the sequences of the existing reference genome [74]. Currently, reference genome sequences of many crops, including rice [75–77], sorghum [78], maize [79], *Brassica rapa* [80], barely [81, 82], millet [83], potato [84], tomato [85], and sesame [14] have been reported. Following the generation of high-quality reference genome sequences, several GWAS have been carried out to discover the natural variation among diverse populations. However, the reference-genome-based GWAS approach may not be sufficient to distinguish any difference between or within the population in which certain relevant genes may be inactive in the reference genome but may be expressed in the studied populations [86].

Since the discovery of pan-genome in *Streptococcus agalactiae* [87], different pan-genomes have been constructed through comparison of multiple genomes derived from *de novo* sequences assembly of various individuals of the same species including, rice [88, 89], maize [90]), soybean [91], *B. napus* [92], wheat [93] and recently in sesame [94] (Table 2). Unlike the reference genome sequencing-based GWAS approach which depends on SNPs among the entire panel under investigation, the pan-genome approach is more inclusive and could detect copious variation including structural variation (SV), copy number variation (CNV), present/absent variation, inversion and translation variations [30, 86]. In this regard, Song et al. [96] reported a direct detection of causal structural variation for the target traits (silique length, seed weight and flowering time) in *Brassica napus* based on the PAV-based genome-wide association study (PAV-GWAS) using the pan-genome assembled from eight high-quality genomes. They also reported that the SNP-GWAS approach that involves the single reference genome indicated no detection of causal structural variation for the same population. The result of their study indicates that the pan-genome based association study is a powerful approach that can complement the single-reference genome approach in detecting new SNP-trait associations. Likewise, the physical position of the sugarcane mosaic virus resistance gene (*ZmTrxh*) in maize was discovered using a pan-genome assembled from three different genotypes, but not with the use of the single reference genome [90]. Other pan-genomes based GWAS have been conducted in important crops such as rice and pigeon pea [89, 97].

**Table 2:** Summary of pan genome assembly in various plant species

Plants	Number of assembled genome	References genome	Pan-genome			
			Number of total genes	% core gene	% of dispensable gene	References
Brassica	21	Darmor-*bzh*	105,672	56	42	[80]
Sesame	5	Zhongzhi13	26,472	58.21	41.79	[94]
Maize	3	B73	59,080	48.6	51.4	[90]
	96	B73	4,400,000	74%	26%	[30]
Rice	66	Nipponbare	42,580	61.94	30.06	[89]
Arabidopsis	18	TAIR10	37,789	69.8	30.2	[95]
Soybean	7	GmaxW82	NA	48.6	51.4	[91]

*NA* data not available

### Diversity and development of GWAS populations in sesame

#### Morphological and genetic diversity

Sesame is a diploid species and belongs to the division *Spermatophyta*, subdivision *Angiospermae*, class *Dicotyledoneae*, order *Tubiflorae*, family *Pedaliaceae*, and genus *Sesamum*. *Pedaliaceae* is a small family of 16 genera and 60 species of which 37 species belong to *Sesamum* genus and only *Sesamum indicum* L. is the most commonly cultivated species [10, 39, 98–100]. A high number of varieties and ecotypes are reported with high adaptation to various ecological conditions in the world. There are three cytogenetic groups in *Sesamum* of which 2n = 26 consists of the cultivated *S. indicum* along with *S. alatum, S. capense, S. schenckii, S. malabaricum*; 2n = 32 consists of *S. prostratum, S. laciniatum, S. angolense, S. angustifolium;* while *S. radiatum, S. occidentale and S. schinzianum* belong to 2n = 64 [101–103]. So far, extensive morphological variations including plant height, height to the first capsule, height to first branch, number of branches, flowering period, flower color, number of flowers per axil, number of capsule per axil, capsule edge number days to maturity, number of seeds per capsule, number of capsule per plant, seed coat color, seed size, seed oil content, seed yield, and branching habit have been reported in the cultivated sesame [11, 14, 104–107]. Besides the huge phenotypic variation harbored in sesame germplasm, various molecular marker-based high levels of genetic diversity were also documented within many landraces and cultivars collected from different areas around the world (Table 3) [1, 14, 15, 104, 106, 109, 110, 115–134]. Recently, advances in next-generation sequencing technologies have facilitated SNP-based genetic diversity analysis in sesame. Globally, high levels of genetic diversity in diverse sesame germplasm from Asia, Europe, America, and Africa were reported (Table 4) [14, 15, 36, 135, 136].

**Table 3:** Summary of molecular marker based genetic diversity and population structure analysis in sesame

Number of accessions	Source of collection	Marker type	Marker size	Detected alleles	Number of allele per locus	PIC	Genetic diversity	Sub populations identified	References
96	Asia and Africa (22 countries)	SSR	33	137	4.15	0.45	0.508	5	[108]
153	Worldwide (22 countries)	SSR	16	121	7.6	0.42	0.46	3	[109]
404	Chinese core collection	SRAP and SSR	14	126	9	0.39	0.24	2	[107]
453	Chinese core collection	SRAD and SSR	14	126	9	0.3467	0.2218	9	[106]
49	India	SSR	20	NA	3	0.718	NA	2	[110]
277	15 countries	SSR	14	158	11.3	0.568	NA	4	[111]
96	China	SSR and InDels	44	113	2.6	0.31	0.37	2	[104]
545	390 from China, 155 outside China	SSR	42	106	NA	0.41	0.645	3	[112]
216	Chinese core collection	SSR, SRAD and AFLP	79	338	2	0.25	0.2090	2	[113]
216	Chinese core collection	SSR, SRAD and AFLP	79	608	2	0.16	0.13	2	[114]
130	China	SSR and InDels	88	325	3.69	0.36	0.432	2	[115]

*PIC* Polymorphism Information Content

**Table 4:** Summary of SNP marker based genetic diversity and population structure analysis in sesame

Number of accessions	Sources of accessions	Number of effective SNPs	SNPs detection approach	Average marker density/SNP	Average nucleotide diversity of the panel	Genetic distance	Average gene diversity	Number of subgroups identified	References
95	Mediterranean sesame core collection (21 geographical regions)	5,292	ddRAD	46SNP/kb	NA	0.023 to 0.524	0.28		[119]
366	HSRC-HAAS	89,924	SLAF-seq	1SNP/2.6kb	1.1×10^-3^	0.01 to 0.42	0.17	3	[122]
705	China gene bank	254,781	Whole-genome sequencing	1SNP/50bp	2.4×10^-4^	0.02		2	[15]
29	China gene bank	127,347	Whole-genome sequencing	NA	1.5 × 10^-4^	NA	NA	NA	[14]

#### Development of GWAS populations

**Fig. 1:**
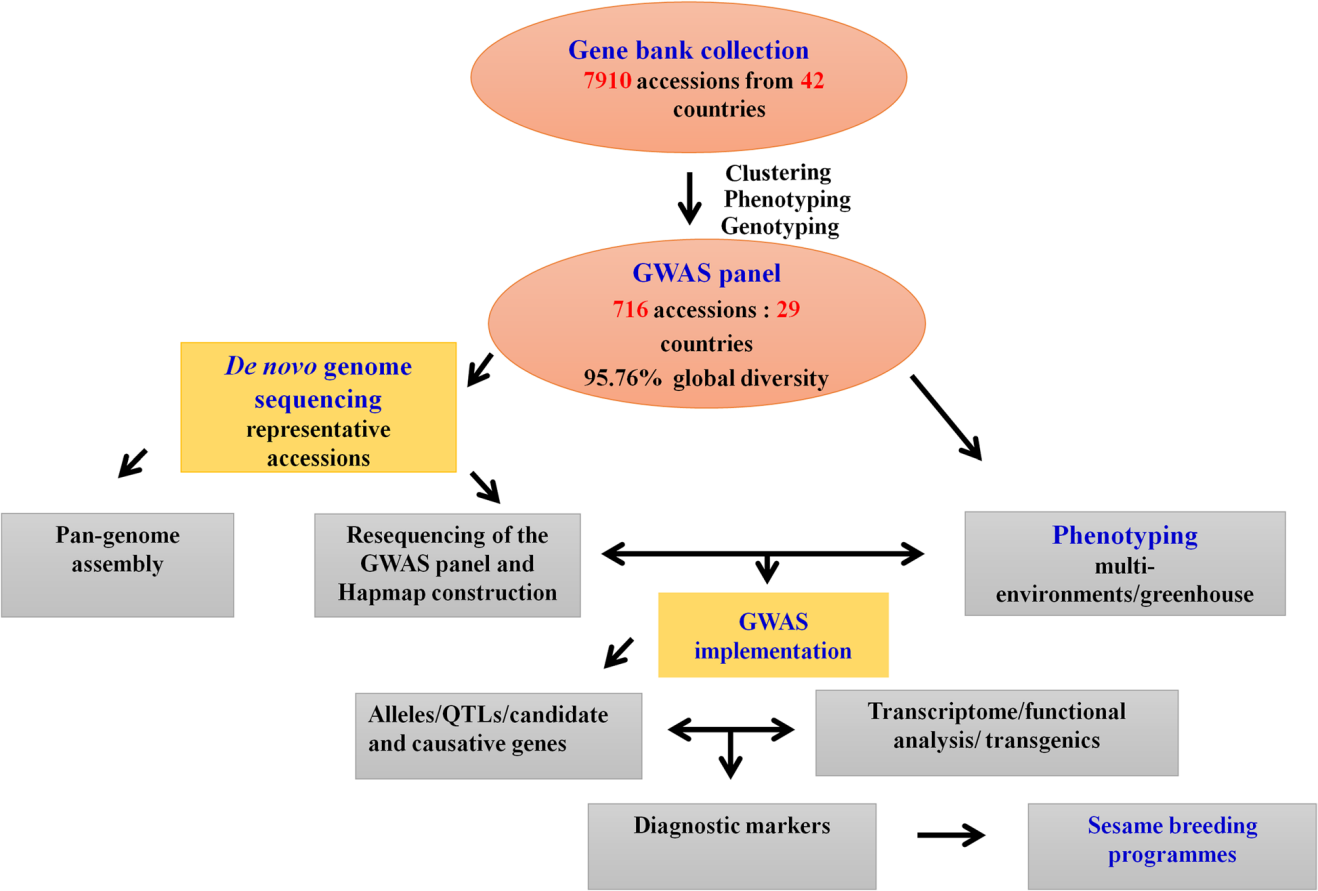
Flow chart showing key steps in GWAS implementation in sesame (prepared based on works at OCRI-CAAS)

**Fig. 2:**
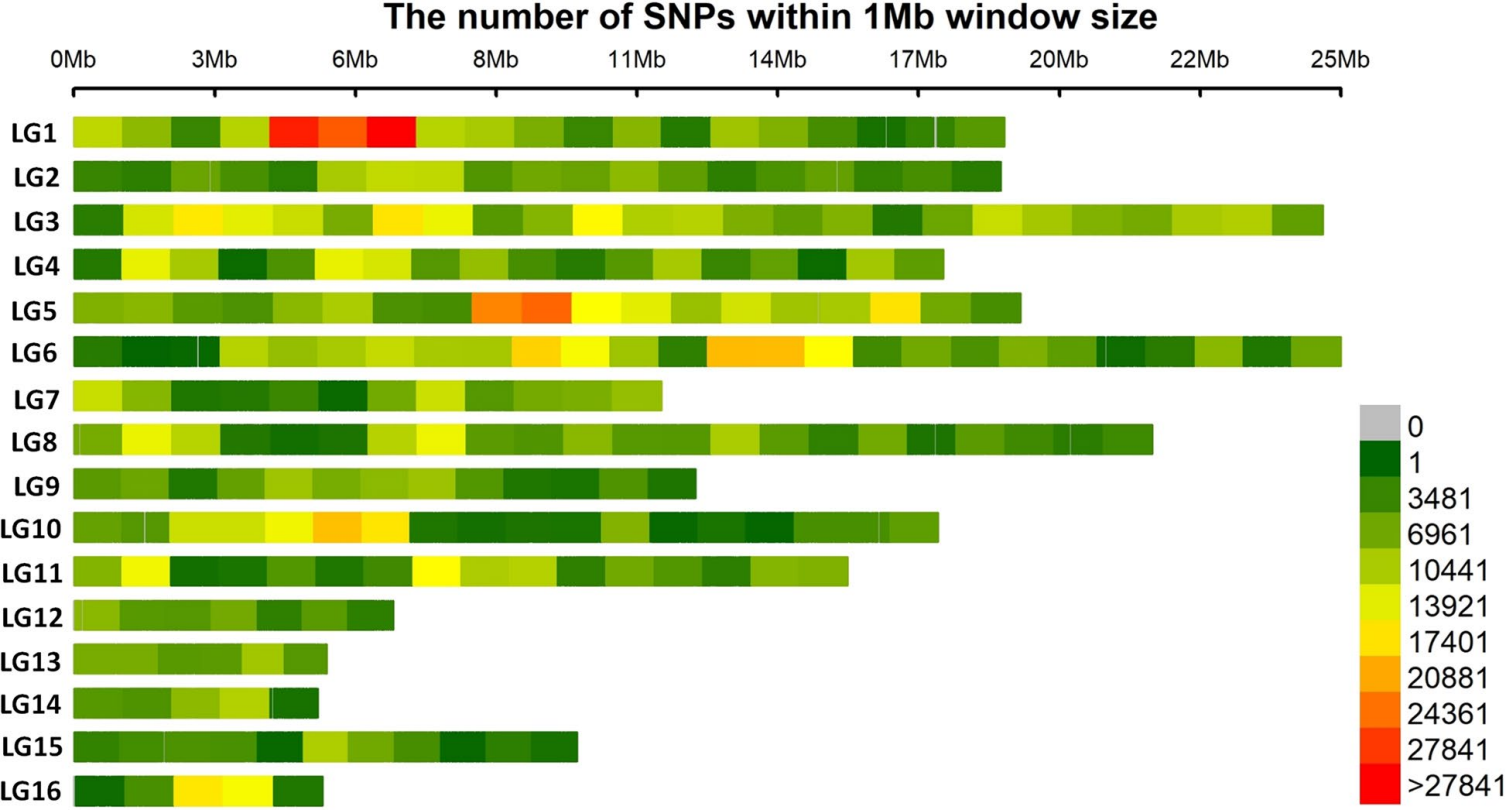
Single-nucleotide polymorphism distributions on the 16 linkage groups (LGs) of the sesame genome assembly v1. The horizontal axis shows the LG length; the 0∼27841 legend insert shows the SNP density

In China, there are over 8,000 accessions of sesame deposited in the National Mid-term Gene Bank of China located in the Oil Crops Research Institute of Chinese Academy of Agricultural Sciences (OCRI-CAAS) [14]. Similarly, about 4,500 sesame accessions conserved in the National Long-term Gene bank in Beijing [107] (Fig. 1). Based on these large collections, strategies to build a sesame core collection have started early in the year 2000 using morphological descriptors and later, molecular tools [14, 15, 106, 107, 137]. Ultimately, a sesame core collection encompassing 705 diverse accessions including 405 landraces, 95 cultivars from China, and 205 accessions from 28 other countries was established at OCRI [15]. The entire panel was re-sequenced on Illumina HiSeq 2000 (http:/www.ncgr.ac.cn/ SesameHapMap), in which a total of 5,407,981 SNPs were detected in the genome with an average of 2 SNP per 50 bp (Fig. 2). This panel shows ideal characteristics for the implementation of GWAS, including high phenotypic variability, low population structure and genetic differentiation among groups, and a moderate decline in LD (~88 kb) [15]. However, most of the accessions (70.1%) included in this panel represent only one country while the other 28 countries are represented only by 29.9% of the accessions. Furthermore, a limited number of African sesame (~3%) was included in this study, although Africa is the main source of diverse sesame landraces [108]. Therefore, for exploiting the genetic bases of important agronomic traits and detection of potential causative genes, there is a need to update this GWAS population panel by including more materials representing diverse agro-ecological origins across the world. Another association-mapping panel population was developed by the sesame research group in Henan Sesame Research Center, Henan Academy of Agricultural Sciences (HSRC-HAAS) [122, 136] consisting of 366 germplasm accessions representing about 89.9% from China and the rest 10.1% from 11 countries. This population also showed high phenotypic and genetic diversity, relatively good SNP density (1 SNP per 2.6 kb with 42,781 SNPs in total) and moderate decay in LD (~99 kb) [122]. However, this panel also has limited geographical representation. Further GWAS panel populations have been recently built from Korean core collections. However, the population size and SNP density were very low: 96 accessions and 5,962 SNPs [36]; 87 accessions and 8,883 SNPs [135]. Overall, to explore the genetic bases of economically important agronomic traits and identify possible causative genes, these developed GWAS panels need to be updated by providing more materials reflecting diverse agro-ecological backgrounds worldwide.

### Advantages and limitations for GWAS implementation in sesame

#### Advantages

Implementation of GWAS based on high-quality genome sequences results generally in a more accurate prediction and mining of potential causative genes. The high-resolution positioning of SNPs in the genome along the entire chromosomes can unravel the genetic architecture of target traits; hence, GWAS can detect more significant associations, candidate genes, and genomic locations with high power and efficiency. Since 2014, the development of a high-quality draft genome of the sesame genotype ‘Zhongzhi13’ [14] has opened the door for genomic research in sesame. Sesame has a small diploid genome estimated at 350 Mb, of which 274 Mb draft genome was assembled, and 27,148 protein-coding genes were predicted. Another genome sequence was also published during the same period from the modern cultivar ‘Yuzhi1’ [138]. Progresses in genome sequencing technologies associated with the reduction of sequencing costs have created opportunities for additional genome sequencing projects in sesame. The reference genome was updated to have a higher resolution [39] and the genome sequences of different sesame landraces including ‘Baizhima’ and ‘Mishuozhima’ [15] and a modern cultivar ‘Swetha’ [139] were also published. Furthermore, the assembly of a sesame pan-genome from five different genomes identified 15,890 dispensable genes, providing a rich resource for comprehensive gene discovery and superior allele mining through GWAS [94]. Similarly, the availability of tremendous transcriptome data from diverse sesame tissues, various growth conditions and from wild *Sesamum* species such as *S. radiatum* and *S. mulayanum* (Table 5) (https://www.ncbi.nlm.nih.gov/bioproject/?term=((sesame)%20AND%20%22Sesamum%20indicum%22[orgn:__txid4182])%20AND%20bioproject_sra[filter]%20NOT%20bioproject_gap[filter]) facilitates post-GWAS works particularly for pinpointing candidate genes and their functional analysis. The availability of several mapping populations [11] is also very useful for validating or polishing GWAS findings. Besides, the availability of functional genomic databases such as Sinbase (http://ocri-genomics.org/Sinbase/index.html), SesameFG (http://sesame-bioinfo.org/SesameFG/) and Sesame HapMap that have been deployed to facilitate genome excavation, comparative genomics, gene expression analysis, are highly useful for post-GWAS investigations [15, 105, 140].

**Table 5:** Summary of RNA-seq data available for various investigated tissues in sesame

Tissue sample	Condition/topic	Sample size	SRA accession numbers
Root	Salt	30	PRJNA524278
Root	Osmotic stress	12	PRJNA552167
Seed and capsule	Seed and carpel development	22	SRR6010084-SRR6010093-SRX396185-SRX396196
Root	Drought	30	SRP095661
Root, leaf, stem and shoot apical	Growth habit		KU240042
Flower buds	Fertile and sterile flower buds	2	SRP095661
Root	Waterlogging	6	SRR2886790
Leaf	Fusarium wilt disease	8	
Leaf, root, stem and flower	Multiple tissues		SRA122023
Seed	Seed developmental stage	12	SRP034617
Seedling	Fusarium wilt disease		SRA047567.1
Seedling	Growth and development	24	SRA047563.1
Seed	Oil content	6	JK045130-JK086377
Root, leaf, flower, developing seed, and shoot tip	Multiple tissues	5	SRP006700

**Fig. 3:**
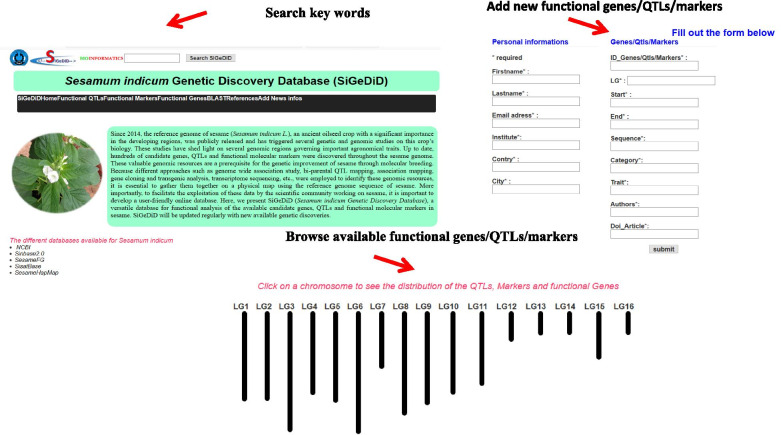
SiGeDiD: an online catalogue of functional genomic discoveries in sesame (http://sigedid.ucad.sn/)

To further facilitate the exploitation of GWAS results as well as all genetic discoveries available in sesame, we have developed a novel database named *Sesamum indicum* Genetic Discovery Database (SiGeDiD) (http://sigedid.ucad.sn/). SiGeDiD is a flexible online catalog of all genetic and genomic discoveries including, candidate genes, QTLs and functional molecular markers in sesame (Fig. 3). It is an essential platform for comparative analysis of GWAS projects in sesame and facilitates gene discovery, particularly the identification of pleiotropic genomic regions/genes that have been identified from different GWAS and other genetic/genomic studies. The website is user-friendly and we integrated a module allowing researchers to upload directly their findings in SiGeDiD. Currently, the BLAST functionality is unavailable but SiGeDiD will be updated to make it more interactive and fully functional.

Collectively, the availability of enormous genomic resources, the small genome size of sesame, comprehensive GWAS panels, diverse mapping populations, high genetic diversity, low population structure, and relatively low LD are advantageous for GWAS implementation in sesame.

### Limitations

While GWAS provides an opportunity to investigate a range of novel genes associated with important agronomic traits, this method does not necessarily identify causal variants and genes [141]. When GWAS is completed, it is often necessary to take additional steps to investigate the functional and causal variants and their target genes in which transgenic experiments may ultimately be implemented. Sesame, however, is a recalcitrant plant for genetic transformation, so there are limited validations of GWAS-identified SNPs using a transgenic approach. Besides, although the LD decay rate in sesame is relatively lower than that of other self-pollinating crops, including rice (~100-350 kb) [142, 143], soybean (~574 kb) [144, 145] and brassica (~405 kb) [146], it showed a higher LD decay rate than other cross-pollinating species, including maize (~5.39-15.53 kb) [147]. Consequently, the modest level of LD decay rate (88 kb) reported in sesame suggests that GWAS resolution may not easily resolve to the causative gene unless a high marker density is used. GWAS, therefore, could have a limited efficiency on trait-based QTL regions or causative genes detection in the absence of high marker density. Another limitation of GWAS in sesame is that many sesame cultivars are highly photosensitive, so field phenotyping and collecting reliable data in various regions of the world is difficult.

### GWAS applications in sesame

**Fig. 4:**
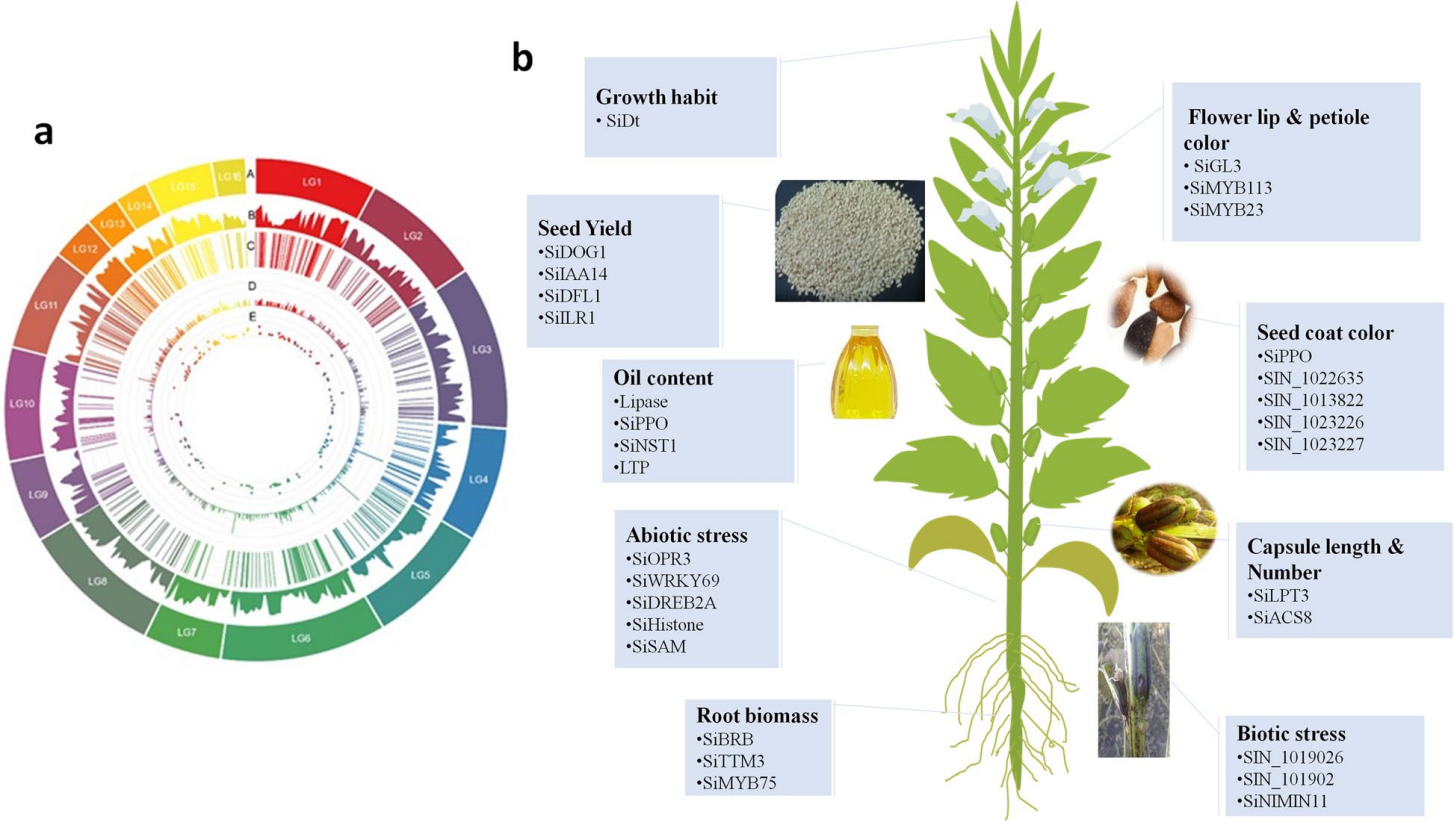
GWAS applications in sesame. **a** Circos plot summarizing genetic findings of important agronomic traits in sesame. (A) Pseudomolecules (LG), (B) gene density, (C) QTL position, (D) -log(p) of the peak SNPs, (E) pleiotropic QTLs; **b** Schematic diagram showing potential candidate genes discovered so far related to important agronomic traits in sesame. The image of the sesame plant has been specifically designed in this study

From 2015, several GWAS projects have been successfully implemented in sesame to uncover the genetic bases of key agronomic traits such as oil content, oil nutrient composition, seed yield, and yield-related components, seed coat color, morphological characteristics, disease resistance salt tolerance, waterlogging resistance, drought tolerance, root traits and nutritional values [15, 33–36, 135, 136, 148]. As to our knowledge, all GWAS projects conducted so far in sesame were based on a single-locus method (EMMA) and the majority was implemented on the GWAS panel developed at OCRI-CAAS. In this work, we summarize all of the results of GWAS reported by different groups of sesame researchers (Table 6 and Fig. 4). A large scale GWAS was conducted by investigating the natural variation of 705 sesame accessions based on 169 sets of phenotypic data including, oil content, nutrient composition, yield components, morphological characteristics, growth cycle, coloration and disease resistance. In total, 1,805,413 SNPs were used. This has led to the identification of 446 significantly associated SNPs with the phenotypic variation. Following in-depth analyses of the major loci, a total of 46 causative genes including genes related to flower lip color (*SiGL3*), petiole color (*SiMYB113* and *SiMYB23*), oil content (*SiPPO*), fatty acid biosynthesis (*CXE17* and *GDSL*-like lipase) and yield (*SiACS*) were identified [15]. Similarly, GWAS of 39 yield-related traits was also conducted [34] using the same population as the previous study [15]. In total, 646 loci associated with traits of interest and 48 potential genes significantly associated with the functional loci were identified. They reported several candidate homologs genes involved in seed formation and some novel candidate genes (*SiLPT3* and *SiACS8*) which may control capsule length and capsule number [34]. Likewise, variations in PEG-induced drought stress and salt stress tolerance were investigated in 490 diverse sesame accessions (representing 33 countries in Asia, Africa, America and Europe) based on GWAS [33]. A total of 132 significant SNPs resolved to nine QTLs and 151 total genes of which *SiEMF1*, *SiGRV2*, *SiCYP76C7*, *SiGRF5*, *SiCCD8*, *SiGPAT3*, *SiGDH2*, *SiRABA1D* were detected as potential genes regulating drought stress while for salt tolerance, a total of 120 significant SNPs resolved to 15 QTLs and 241 genes of which of *SiLHCB6*, *SiMLP31*, *SiPOD*, *SiHSFA1*, *SiDUF538*, *SiCC-NBS*-LRR, *SiUDG*, *SiGPAT3*, *SiNAC43*, *SiGDH2*, *SiCP24*, *SiWRKY14*, *SiXXT5*, *SiXTH15*, and *SiG6PD1* were detected as potential genes [33]. Later on, GWAS was conducted to investigate genetic variants governing drought tolerance in 400 sesame accessions [35]. A total of 140 reliable and stable QTLs were identified and resolved to 10 QTLs. Similarly, 120 genes, of which *SiABI4*, *SiTTM3, SiGOLS1*, *SiNIMIN1*, and *SiSAM* having high potentials to modulate drought tolerance in sesame, were identified [35]. Their study was the first to validate the function of a candidate gene from GWAS using transgenic approach. They demonstrated that sesame accessions originated from drought-prone agro-ecological regions have fixed several drought-tolerant alleles, though alleles contributing to high yielding under drought conditions are far from being fixed. Hence, sesame is mostly considered as a resilient crop because of the long-term adaptation to drought-prone agro-ecological regions. Additional new GWAS results were also reported recently [36, 135, 136] (Table 6). Based on genotyping by sequencing (GBS) method, [36] conducted GWAS on vitamin E and identified eight strongly linked SNPs and 12 genes with various regulatory functions, including transcription regulator HTH, zinc ion binding protein, glycosylphosphatidylinositol (GPI)-anchor biosynthesis and ribosome protein. They also identified, two loci, LG_03_13104062 containing seven genes (*SIN_1022039*–*SIN_1022045*) and LG_08_6621957 containing five genes (*SIN_1001936*–*SIN_1001940*), detected simultaneously on LGs 3 and 8, respectively, by employing two different models (GLM and MLM). Hence, the authors suggested that these two simultaneously detected loci have high potentials to control vitamin E in sesame. However, due to the limited numbers of SNPs (5,962) and small panel size used in this GWAS, potential loci for this important trait may have been missed [136]. used genotype data from 42,781 SNPs and seed coat color trait from an association-mapping panel consisting of 366 sesame germplasms to identify 224 significantly associated SNPs. Based on the four most stable peaks/SNPs significantly associated with sesame seed coat color, they retained 92 candidate genes. Of these genes, *SIN_1016759* (encoding predicted PPO) was also reported in previous GWAS by [15] and QTL mapping study by [39]. Using a mapping association of 87 sesame accessions and 8,883 SNPs, a GWAS on phytophthora blight resistance was conducted [135]. The result of this study suggested that *SIN_1019016* was one of the candidate genes identified closely associated with phytophthora blight resistance in sesame. The limited SNP numbers called from the GBS approach and relatively small size of sesame accessions used in this study could have affected the GWAS output associated with trait under investigation. More recently, a comprehensive GWAS conducted by Dossa et al. [148] unraveled the genetic basis of seven root related traits. They reported 409 significant signals, 19 QTLs containing 32 candidate genes associated with sesame root traits. More importantly, they discovered an orphan gene named ‘*Big Root Biomass*’ (*SIN_1025576*) which modulates sesame root biomass through the auxin pathway [148]. In addition to the published GWAS findings, the OCRI-CAAS sesame research group has also several unpublished GWAS outputs on various agronomic traits including, waterlogging, chlorophyll, salt stress at the seedling stage and interestingly a metabolite based GWAS has been completed. These results will illuminate the genetic basis of important metabolites such as sesamin/sesamolin variation in sesame. All candidate genes, QTLs and SNPs will be regularly loaded into SiGeDiD (http:/sigedid.ucad.sn/) for further uses in sesame breeding projects.

**Table 6:** Summary for GWAS results reported so far in sesame

Sample size	Targeted traits	Group of traits	SNP size	SNP density	Significant associations identified	Number of candidate genes	Potential genes	References
705	56	Oil content, nutrient composition, yield components, morphological characteristics, growth cycle, coloration and disease resistance	1,805,413	1SNP/50bp	446	46	- *SiGL3* ~flower in lip colour - *SiMYB113* & *SiMYB23* ~ petiole colour - *SiPPO* ~oil content, seed coat color, protein content - *SIN_1016759* encodes a predicted PPO ~seed coat color - *CXE17* & GDSL-like lipases ~ encoding lipase - *SIN_1019167* & *SIN_1009923* ~encoding lipid transfer protein - *SiACNA*, *SiDGAT2*, *SiFATA*, *SiFATB* and *SiSAD*~ fatty acid composition - *SiFAD2* ~ oleic acid desaturase - *SiACS*~ seed yield	[15]
705	39	Yield index, seed traits, capsule number, capsule size, and capsule pericarp	1,805,413	1SNP /50bp	646	48	- *SiACS8* ~capsule number - *SiLPT3 ~* capsule length	[34]
490	4	Drought and salt tolerances	1,005,413	2,7SNP / kb	252	40	- *SiOPR3* ~ increase of abscisic acid during desiccation - *SiWRKY69* ~ functioning in response to dehydration stress - *SiCCD8* ~ functions as a carotenoid cleavage dioxygenase - *SiMLP31*~ salicylic acid synthesis - *SiANTH* ~ phosphatidic acid-binding protein - *SiHKT1* ~ sodium transporter - *SIN_1021330, SIN_1021327, SIN_1021326, SIN_1021325, SIN_1021324, SIN_1021323* and *SIN_1021322*~ encoding sesame peroxidase	[33]
400	5	Drought (stem length, survival rate, wilting level, capsule number and seed yield)	1,000,939	5SNP /kb	569	102	- *SiABI4 ~* involved in abscisic acid signal transduction - *SiTTM3, SiGOLS1, SiPOD3 & SiNIMIN1 ~* involved in drought tolerance - *SiSAM* ~ modulates polyamine levels	[35]
96	1	Vitamin E	5,962	2.3SNP /100 kb	8	12	- LG08_6621957 loci ~ γ-tocopherol - SLG03_13104062 loci ~β-tocotrienol	[36]
87	1	Phytophthora disease-resistant	8,883	NA	44	68	- *SIN_1019026 & SIN_1019021~ regulation* of pathogen-induced signaling - *SIN_1019014* & *SIN_1018999*~ encoded F-box - *SIN_1019019 & SIN_1018986~* cytochrome P450 family protein - *SIN_1019026 & SIN_1019021~* encoding ubiquitin ligases and ubiquitin-related modifiers	[135]
366	1	Seed coat color	42,781	1SNP/2.6kb	224	92	- *SIN_1016759* ~ PPO - *SIN_1023237* ~ laccase3 - *SIN_1006022* ~ cytochrome P450 - *SIN_1023226* & *SIN_1024895* ~ WRKY and *bHLH130*	[136]
327	7	Root traits	1,000,000	5SNP /kb	409	32	- *SiBRB*	[148]

### Potential of new statistical models to improve the accuracy and power of GWAS in sesame

To our knowledge, multi-locus models have not yet been employed in sesame GWAS research and no previous study has compared different GWAS models (single locus and multi-locus models) in sesame. Herein, we tested the applications of new GWAS models in sesame based on quantitative (root length) and qualitative (seed coat color) traits. Natural variation in root length of 350 sesame accessions was collected from a field experiment following the methodology developed by Su et al. [149], and the genotypic data were obtained from 1,000,000 common SNPs. For the seed coat color GWAS, the 600 sesame accessions, and 1,000,000 common SNPs were used [15]. To investigate the phenotypic natural variation for the seed coat color, matured seeds from five capsules per genotype were collected and photographed with a high-resolution digital camera and the seed –coat color data, which was based on the red, green, and blue (RGB) values, were recorded following the methodological approach adopted by Zhang et al. [150]. Subsequently, three separate GWAS models, including two multi-locus models (mrMLM FASTmrEMMA and mrMLM ) and one single locus model (EMMAX) were selected (mainly because they do not require extensive phenotypic and genotypic data formatting) and were implemented using the phenotypic and genotypic data. We further compared the results of these three models to evaluate their potentials to reveal higher number of marker-trait associations and discover more candidate genes.

**Fig. 5:**
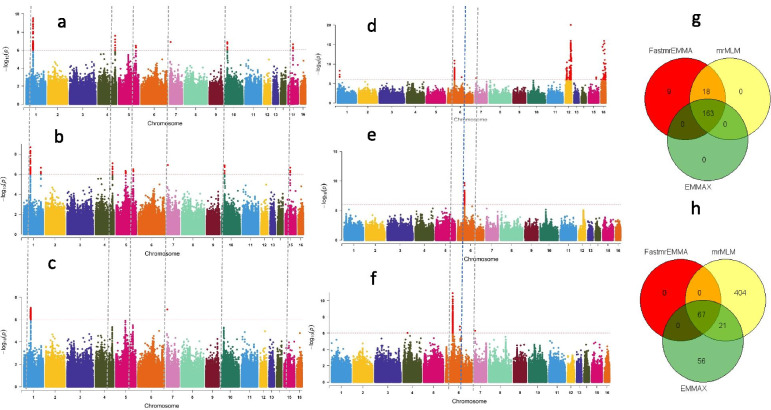
Application of new statistical multi-locus models in sesame. **a** and **b** Negative log10 *P*-values for association of root length (Y-axis) are plotted against SNP positions (X-axis) using the multi-locus models, mrMLM and FASTmrEMMA, respectively; **c** Negative log10 *P*-values for association of root length (Y-axis) are plotted against SNP positions (X-axis) using the single-locus model, EMMAX; **d** and **e** Negative log10 *P*-values for association of seed coat color (Y-axis) are plotted against SNP positions (X-axis) using the multi-locus models, mrMLM and FASTmrEMMA, respectively; **f** Negative log10 *P*-values for association of seed coat color (Y-axis) are plotted against SNP positions (X-axis) using the single-locus model, EMMAX. For both traits, a horizontal dash–dot line indicates the significant *P*-value threshold (10^-6^) and the significant SNPs are highlighted by red color, vertical line indicates overlapped most significant peaks at least in two models; **g** Venn diagrams showing the shared and uniquely detected significant SNPs by each model for root length GWAS respectively; **h**, Venn diagrams depicting the shared and uniquely detected significant SNPs by each model for seed coat color GWAS. The phenotypic and genotypic data for this analysis were obtained from 350 sesame accessions and 1,000,000 common SNPs for root length and data from 705 sesame accessions and 1,805,413 common SNPs for seed coat color GWAS study

Our GWAS results for the two traits showed that a total of 190, 181 and 162 significant SNPs (-log10(p) > 6) associated with root length were detected by FASTmrEMMA, mrMLM and EMMAX, respectively. Similarly, 67, 492 and 143 significant SNPs associated with seed coat color were detected by FASTmrEMMA, mrMLM and EMMAX, respectively (Fig. 5a-f; Table 7; Table S1). Of the significant SNPs associated with root length, 163 SNPs were identified simultaneously by all three models; all the SNPs identified by EMMAX were also identified simultaneously by both multi-locus models, while 18 SNPs were simultaneously and only detected by FASTmrEMMA and mrMLM (Fig. 5g). For the seed coat color associated SNPs, 67 and 27 SNPs were detected by all the three models and by two models (mrMLM and EMMAX), respectively (Fig. 5h). By considering all SNPs co-clustered with peak SNPs within a window of 200 kb as QTLs [35], a total of 19 and 34 QTLs were detected for root length and seed coat color, respectively, by all the three models (Table S1). Within these QTLs, we retrieved 26 and 47 genes for root length and seed coat color, respectively. Based on the robust QTLs co-detected by different models identified for root length, nine potential candidate genes, including *SIN_1017810*, *SIN_101781, SIN_1017812*, *SIN_1017815*, *SIN_1017843*, *SIN_1007064*, *SIN_1007065, SIN_1020072* and *SIN_1017818* are proposed for further functional studies to pinpoint the causative gene (s). Regarding the seed coat color, the potential candidate genes identified in our study include *SIN_1007188*, *SIN_1007221*, *SIN_1023226, SIN_1023227* and *SIN_1023228*. Interestingly, three genes detected in this study were previously reported by Mei et al. [136].

**Table 7:** Summary of significant SNPs associated with root length and seed coat color within the linkage groups (LG) identified by each model during GWAS in sesame

Trait	LG	GWAS models		
		mrMLM FASTmrEMMA	mrMLM	Emmax
**Root length**	LG1	167	163	162
	LG4	7	6	0
	LG5	7	3	0
	LG7	1	1	1
	LG10	5	5	0
	LG15	3	3	0
	**Total**	**190**	**181**	**163**
**Seed coat color**	LG1	0	4	0
	LG4	0	0	0
	LG6	67	89	142
	LG7	0	1	1
	LG12	0	349	0
	LG16	0	48	0
	**Total**	**67**	**491**	**143**

Collectively, the analysis of different GWAS models indicates the potential of using an integrated approach (single and multi-locus models) to improve the capacity and power of GWAS in sesame. This will help to detect more and novel marker-trait associations and candidate genes, particularly when investigating quantitative traits**.** It is also important to note that significantly associated regions simultaneously detected by more models in GWAS are more likely to be highly associated with the traits under investigation as compared with regions detected only by a single model. Hence, developing diagnostic markers for the co-detected associated regions could speed up sesame molecular breeding programmes.

## Conclusions

Over the last five years, GWAS have been successfully implemented in sesame and is illuminating the genetic basis of many important agronomic traits. Even though a list of QTLs (~300) and candidate genes (~250) have been identified for qualitative and quantitative traits, more traits, including chlorophyll-yield, metabolite-GWAS, waterlogging, heat tolerance are under investigation. We envision that all these results will lead to the development of allele-specific diagnostic markers to be used as daily molecular tools in sesame breeding programmes. Though a high-quality sesame reference genome sequence has been developed, more often, there are limitations to find any candidate gene around the peak SNPs from GWAS. To overcome these limitations, we need to use the recently developed sesame pan-genome [94] for future GWAS implementations. The diversity of recently available sesame GWAS panels should be improved by integrating more accessions and wild species from different agro-ecological origins mainly from Africa. For this, an international collaboration between sesame researchers is highly required. Furthermore, collaboration between researchers for generating comprehensive germplasm characterization data using precise phenotyping platforms and in contrasting environments will permit more accurate dissection of the genetic architecture of complex traits in sesame. Efforts towards sharing genetic materials between research institutes are crucial for accelerating gene discovery. For example, the re-sequencing data of the 705 fully sequenced GWAS panel generated by OCRI is publicly available and if the germplasm, at least partly, could be shared with partners, more GWAS projects could be implemented on sesame, particularly on traits highly affected by environments. Similarly, working to develop an SNP chip can be an alternative for quick, low-cost, and easy genotyping of novel sesame collections to be used for future GWAS projects.

The application of new multi-locus GWAS models and integration of single- and multi-locus models will provide more efficiency and power in future GWAS implementation in sesame. Up to date, very few studies have validated the numerous GWAS findings in sesame. Therefore, follow-up studies are needed for further validating the favorable alleles identified from GWAS in independent populations and using other approaches (classical bi-parental QTL mapping, QTLseq, etc.). Validation of GWAS findings using transgenic approach is also instrumental in several plant species. In sesame, genetic transformation protocols using tissue culture techniques have been reported [151]. More studies on this topic are needed in order to develop a more effective genetic transformation protocol in sesame, for example using the flower dip technique [152]. Hairy root genetic transformation is also a flexible and rapid technique widely adopted in several recalcitrant plants to study gene functions [153]. We propose to develop a hairy root genetic transformation protocol in sesame combined with new genome editing technologies to confirm some important GWAS findings. Finally, projects aiming at developing diagnostic molecular markers based on GWAS peak SNPs and their favorable alleles should be instigated. This will considerably accelerate sesame molecular breeding.

## Supplementary Information

**Additional file 1 **: **Table S1**. Summary list of total QTLs and candidate genes identified in GWAS for root length and seed coat color along the linkage groups in sesame by multi-locus and single-locus models. **Table S2**. Summary of QTL and candidate genes detected by each GWAS model. **Table S3**. Candidate genes detected in each LG for each model.

## Data Availability

The data used in this review article are available in the supplementary files and within the manuscript.

## Abbreviations

GWAS: Genome wide association study
LD: Linkage disequilibrium
QTL: Quantitative trait locus
QTN: Quantitative trait nucleotides
SiGeDiD: *Sesamum indicum* genetic discovery database
SNP: Single nucleotide polymorphism
